# Effects of metformin on Sonic hedgehog subgroup medulloblastoma progression: *In vitro* and *in vivo* studies

**DOI:** 10.3389/fphar.2022.928853

**Published:** 2022-10-07

**Authors:** Huangyi Fang, Lingfei Wang, Lisheng Yu, Fang Shen, Zelin Yang, Yue Yang, Shize Li, Haipeng Dai, Feng Tan, Jian Lin, Hansong Sheng

**Affiliations:** ^1^ Department of Neurosurgery, The Second Affiliated Hospital of Wenzhou Medical University, Wenzhou, China; ^2^ The Second School of Medicine, Wenzhou Medical University, Wenzhou, China; ^3^ Department of Surgery, Box Hill Hospital Eastern Health, VIC, Australia; ^4^ School of Basic Medical Sciences, Wenzhou Medical University, Wenzhou, China

**Keywords:** medulloblastoma, metformin, Sonic hedgehog signaling pathway, AMPK, anticancer

## Abstract

Metformin is a first-line drug for type 2 diabetes, and its anticancer effects have also been widely studied in recent years. The Sonic hedgehog (Shh) signaling pathway is involved in the initiation and progression of medulloblastoma. In order to develop a new treatment strategy for medulloblastoma (MB), this study investigated the inhibitory effect of metformin on MB and the underlying mechanism of metformin on the Shh signaling pathway. The effect of metformin on proliferation was evaluated by the cell counting kit-8 (CCK-8) test and colony formation experiment. The effect of metformin on metastasis was assessed by the scratch-wound assay and transwell invasion assay. Cell cycle and apoptosis were evaluated by flow cytometry, and the associated proteins were examined by western blotting. The mRNA and protein expression levels related to the Shh pathway were measured by quantitative PCR, western blotting, and immunofluorescence staining. The xenograft murine model was carried out to evaluate the anticancer effect of metformin on medulloblastoma *in vivo*. Metformin inhibited proliferation and metastasis of the Shh subgroup MB cell line, and the inhibitory effect on proliferation was related to apoptosis and the block of the cell cycle at the G0/G1 phase. Animal experiments showed that metformin inhibits medulloblastoma growth *in vivo*. Moreover, metformin decreased mRNA and protein expression levels of the Shh pathway, and this effect was reversed by the AMP-activated protein kinase (AMPK) siRNA. Furthermore, the pro-apoptotic and cell cycle arrest effects of metformin on Daoy cells could be reversed by the Shh pathway activators. Our findings demonstrated that metformin could inhibit medulloblastoma progression *in vitro* and *in vivo*, and this effect was associated with AMPK-mediated inhibition of the Shh signaling pathway *in vitro* studies.

## Introduction

MB is the most common malignant brain tumor in children, accounting for about 20% of all brain tumors in children under 15 years of age (Patel et al., 2014). MB has the characteristics of high malignancy, rapid growth, easy metastasis, and easy recurrence, and the 5-year survival rate is only 50%–75% (Northcott et al., 2019). The comprehensive treatment of MB patients with surgery, radiotherapy, and chemotherapy is effective for most patients with medulloblastoma. Still, most survivors endure the adverse effects of radiotherapy and chemotherapy for a long time, including developmental, neurological, and endocrine misalignment (Yeole et al., 2021).

With the development of molecular diagnostics, MB has been divided into four subgroups (Wnt, Shh, Group 3, and Group 4) (Chatterjee et al., 2022). The Shh subtype accounts for about 30%, which is more common in infants and adults and has a poor prognosis. In most cases, the Shh subgroup involves somatic mutations in one or more genes of the Shh pathway (such as Ptc, Sufu, or Smo, etc.), leading to abnormal activation of the pathway and further leading to the occurrence of MB((Amayiri et al., 2021), (Skowron et al., 2021)).

Metformin is an oral hypoglycemic drug for treating type 2 diabetes, which mainly acts through AMPK (de Marañón et al., 2022). Previous clinical observational studies have found that taking metformin can significantly reduce the incidence and mortality of various cancers, including gastric cancer, thyroid cancer, and prostate cancer (Kim et al., 2014; Heckman-Stoddard et al., 2017; Park et al., 2018). In the study of prostate cancer and breast cancer, it was also found that activation of AMPK mediates the anticancer effects of metformin (Fan et al., 2015; Chen et al., 2021). In recent years, studies have found that AMPK has a direct and indirect regulatory impact on the Shh pathway. In this study, we selected the Daoy and ONS-76 cell lines (Shh pathway-activated medulloblastoma) as the research object (Azatyan et al., 2021). We examined the inhibitory effect of metformin on MB and identified its association with the AMPK/Shh signaling pathway.

## Methods

### Chemicals

Metformin was ordered from Sigma-Aldrich (St. Louis, MO, United States). Fetal calf serum (FBS), RPMI 1640 medium, and 0.25% Trypsin-EDTA were purchased from Gibco (Grand Island, NY, United States). Smoothened Agonist SAG was purchased from Abcam (Cambridge, MA, United States). The small interfering RNA (siRNA) specific to AMPK (sc-45312) and normal control (sc-37007) came from Santa Cruz (CA, United States). The CCK-8 was purchased from Dojindo Chemical Research Institute (Tokyo, Japan). 0.5% crystal violet was ordered from Sigma-Aldrich (St. Louis, MO, United States). The primary antibody of Caspase-3 (AF6311), Cleaved-caspase-3 (Asp175) (AF7022), Bax (AF0120), Bcl-2 (AF6139), Cyclin B1 (AF6168), Cyclin D1 (AF0931), Cdk4 (DF6102), Shh (DF7747), Ptc (AF5202), Smo (DF5152), Sufu (DF7687), Gli-1 (DF7523), AMPK alpha (AF6423), Phospho-AMPK alpha (Thr172) (AF3423), and β-actin (AF7018) for western blot was obtained from Affinity Biosciences (Cincinnati, OH, United States). All primary antibodies were used at a ratio of 1:1,000 in western blot assays.

### Cell culture

The Daoy and ONS-76 cell lines were obtained from American Type Culture Collection (ATCC, Manassas, VA, United States). The Daoy and ONS-76 cells were maintained in RPMI 1640 medium supplemented with 10% FBS and 1% penicillin–streptomycin (HyClone, Logan, UT, United States). The cells were maintained at 37°C with 5% CO_2_ under the condition of a humidified atmosphere.

### RNA interference

To transfect Daoy cells, the AMPKα1 siRNA or control siRNA was used with Lipofectamine 3,000 Transfection Reagent (Invitrogen, Carlsbad, CA, United States), according to the manufacturer’s protocol. The cells were treated in a serum-free medium for 48 h after transfection, then used in the following experiments.

### Cell proliferation analysis

The CCK-8 assay was performed to detect cell proliferation according to the instructions of the kit. The Daoy and ONS-76 cells were seeded into a 96-well plate at a density of 8 × 10^3^ cells/well and treated separately with different concentrations of metformin and SAG (100 nm) after cell attachment. Subsequently, 10 μL CCK-8 and 100 μL fresh RPMI 1640 medium solution were added to each well and incubated for 1 h. The absorbance at 450 nm was measured with a microplate reader (Thermo Fisher Scientific, Waltham, MA, United States). For the colony formation experiment, Daoy and ONS-76 cells were trypsinized, and 500 viable cells were seeded into 6-well plates. After 24 h, the cells were treated with metformin at a concentration of 0, 1, 3, and 9 mm. After 7 days, the cells were fixed and dyed with 0.5% crystal violet staining (Sigma-Aldrich, St. Louis, MO, United States). Finally, the number of visible colonies was counted.

### Scratch assay

A scratch was made on the top center of cells using a sterile plastic 200-μL micropipette tip. And the medium was replaced with a serum-free medium. Cell migration at 0, 12, 24, and 48 h after scratching was recorded with a microscope. We used Adobe Photoshop (Adobe Systems, San Jose, CA, United States) to measure the scratch with to calculate the migration rate.

### Cell invasion assay

Daoy cells were seeded into the 6.5 mm Matrigel-coated (BD Biosciences, San Jose, CA, United States) Transwell inserts (Corning Costar Corp, Cambridge, MA, United States) at a density of 3 × 10^5^ cells per insert. RPMI 1640 medium with 20% FBS and pure RPMI 1640 medium without FBS were respectively placed in the lower and upper chambers. Following 24 h of treatment with different concentrations of metformin, the cells in the lower chamber were fixed with 4% paraformaldehyde and stained with 0.5% crystal violet. Then the cell images were captured and counted at ×200 magnification using a Nikon Eclipse E 400 microscope (Nikon, Fukuoka, Japan). Finally, we used ImageJ (National Institutes of Health, Bethesda, MD, United States) to measure the cell number.

### Apoptosis analysis

Daoy cells were seeded into a 6-well plate (1 × 10^6^ cells/well) and treated separately with different metformin concentrations and SAG (100 nm) for 24 h. The cells were trypsinized, then washed with phosphate-buffered saline (PBS). We used the Annexin V-fluorescein isothiocyanate (FITC) kit (Beyotime Institute of Biotechnology) to quantify apoptosis, according to the instructions. The cells were resuspended in a binding buffer and stained with Annexin V-FITC/propidium iodide (PI). Cell apoptosis was examined by flow cytometry (Thermo Fisher Scientific, Waltham, MA, United States). Data analysis was analyzed using the FlowJo software (Tree Star Inc, Ashland, OR).

### Cell cycle analysis

Daoy cells were seeded into a 6-well plate (1 × 10^6^ cells/well) and treated separately with different metformin concentrations and SAG (100 nm) for 24 h. The cells were trypsinized, then washed with PBS. The cells were centrifuged and fixed with ice-cold 70% ethanol at 4°C overnight. The cells were incubated with RNase (Dojindo, Kumamoto, Japan) and PI in an incubator at 37°C for 30 min (min) on the second day. The cell cycle distribution was examined by flow cytometry (Thermo Fisher Scientific, Waltham, MA, United States). Data analysis was analyzed using the FlowJo software.

### Quantitative PCR

Daoy cells were seeded into a 6-well plate (1 × 10^6^ cells/well) and treated with different metformin concentrations for 24 h. Total cellular RNA was extracted with TRIzol reagent (Invitrogen) according to the manufacturer’s instructions. The total RNA extracted was then reverse-transcribed into cDNA with the PrimeScript RT Master Mix. The primers specific for each molecule were designed to generate the PCR products. The following primers were used: Shh-Forward: 5′-CGC​ACG​GGG​ACA​GCT​CGG​AAG​T-3'; Shh-Reverse: 5′-CTG​CGC​GGC​CCT​CGT​AGT​GC-3'; Smo-Forward: 5′-TTA​CCT​TCA​GCT​GCC​ACT​TCT​ACG-3'; Smo-Reverse: 5′-GCC​TTG​GCA​ATC​ATC​TTG​CTC​TTC-3'; Ptc-Forward: 5′-TCT GCA​GCA​ACT​ATA​CGA​GC-3'; Ptc-Reverse: 5′-GAACAGCTCGACC GTCATCA-3'; Gli-1-Forward: 5′-GGA​CAA​CCG​CCA​TCC​AGA​CT-3'; Gli-1-Reverse: 5′-GCC​AGG​GAC​ACC​TCC​ATC​TC-3'; GAPDH-Forward: 5′-TCA​CCA​TCT​TCC​CAG​GAG​CGA​G-3'; GAPDH-Reverse: 5′-TGT​CGC​TGT​TGA​AGT​CAG​AG-3'. The samples were examined with a PCR array (Takara, Japan). Data were analyzed by the 2^−ΔΔCT^ method.

### Western blot analysis

The cells are properly treated and placed on ice before adding RIPA buffer containing protease inhibitor cocktail (ratio, 100:1) (Roche Diagnostics Corp. Indianapolis, IN, United States). The protein concentration was examined by the BCA method (ab102536; Abcam), separated by 8%–12% sodium dodecyl sulfate–polyacrylamide gel electrophoresis, then transferred to a polyvinylidene difluoride membrane (Abcam, Cambridge, MA, United States). Subsequently, the membrane was blocked with 5% non-fat milk in Tris-buffered saline containing Tween-20 (TBST) for 90 min at room temperature. Then the membrane was washed with TBST and incubated at 4°C overnight with the primary antibody in a ratio of 1:1,000. The next day, the membrane was washed with TBST and followed by incubation with horseradish peroxidase-coupling secondary antibody (goat anti-rabbit) at room temperature for 2 h. At last, the membrane was washed with TBST and detected with BeyoECL Plus developer (Beyotime, Shanghai, China) using the Bio-Rad Molecular Imager FX.

### Immunofluorescence staining

Daoy cells were treated separately with metformin (3 mm) and SAG (100 nm) for 24 h, fixed in 4% paraformaldehyde for 15 min at room temperature and then permeabilized with 0.2% Triton X-100 for 20 min. After blocking for 30 min in 10% goat serum, the cells were incubated with the primary antibodies Gli-1 (1:200 dilution) overnight at 4°C. Then, the cells were washed three times with PBS and incubated with AlexaFluor 488 (goat anti-rabbit IgG, Abcam, Cambridge, UK, 1:1,000 dilution) for 1 h at room temperature. Finally, the cells were stained with Dapi (1 μg/ml) for 10 min and imaged using an inverted IX71 microscope system (Olympus, Tokyo, Japan). The mean intensity was measured by ImageJ software.

### Tumor xenografts in nude mice

Daoy cells (5 × 10^6^) were resuspended in PBS and injected subcutaneously into 6-week-old BALB/c nude mice (Shanghai Laboratory Animal Center, Shanghai, China). About 10 days later, the mice were randomly assigned to two groups (control group and metformin group). The mice in the control group were orally administrated with 300 μL PBS daily, while the mice in the metformin group were orally administrated with 300 μL metformin (200 mg/kg) daily. The mice were measured for body weight and tumor volume every 3 days. The tumor volumes were measured using a vernier caliper and calculated as 0.5 × length × width^2^. After 24 days of treatment, the tumors were removed from the mice, weighed, and photographed. All mouse studies were carried out according to the institutional guidelines for the use of animals, and all procedures were approved by the Ethics Committee of the Second Affiliated Hospital of Wenzhou Medical University.

### Statistical analysis

Statistical results were analyzed with GraphPad Prism 8.00 (GraphPad Software, Version X; La Jolla, CA, United States). All experimental data were shown as mean ± standard deviation. Data were statistically analyzed by Student’s t-test, one-way analysis of variance (ANOVA), or two-way ANOVA. *p* < 0.5 was considered statistically significant.

## Results

### Metformin inhibited the growth, migration, and invasion of the Shh subgroup MB cell line

**FIGURE 1:**
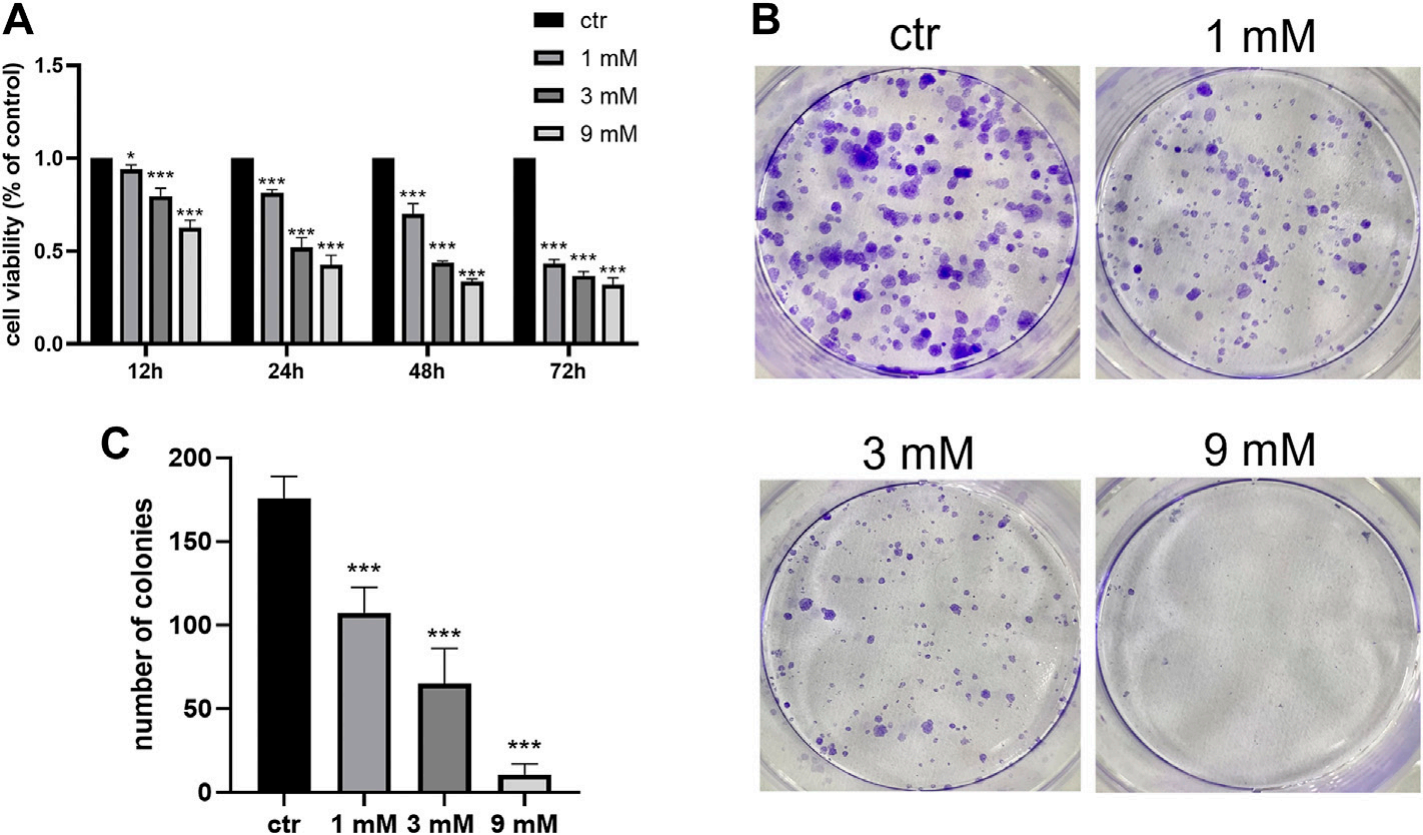
Metformin inhibited the proliferation of Daoy cells. **(A)** CCK-8 assay was used to investigate the effects of metformin treatment on cell viability (*n* = 6). **(B,C)** Colony formation assay was used to detected the effects of metformin treatment on clone ability of cells (*n* = 3). Data are presented as the mean ± SD, t-tests were used to determine the significance. *p < 0.05; ***p < 0.001 compared with the control group.

**FIGURE 2:**
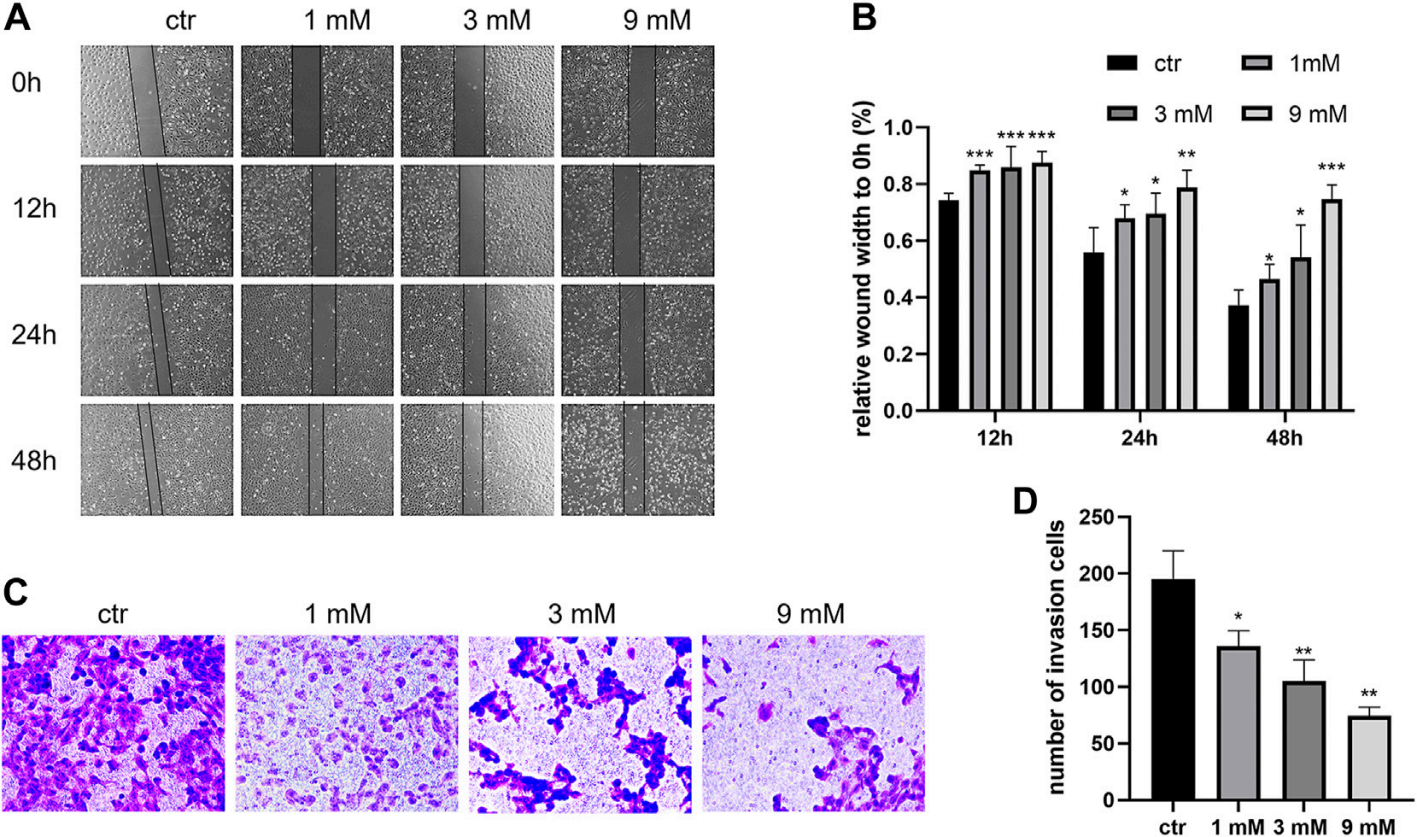
Metformin suppressed the migration and invasion of Daoy cells. **(A,B)** Scratch assay was used to assess the migration ability of Daoy cells after metformin treatment (*n* = 4). **(C,D)** Transwell assay was used to assess the invasion ability of Daoy cells after 24 h of metformin treatment (*n* = 3). Data are presented as the mean ± SD, t-tests were used to determine the significance. *p < 0.05; **p < 0.01; ***p < 0.001 compared with the control group.

To examine the anticancer effect of metformin in Shh subgroup MB, cell viability was detected by the CCK-8 assay. After treating Daoy cells with metformin (0, 1, 3, and 9 mm) for 12 h, 24 h, 48 h, and 72 h, we found the cell viability decreased with the increase in metformin concentration and treatment time (Figure 1A). The results of the colony formation experiment showed that the number and size of the colonies decreased with the increase in metformin concentration (Figures 1B and C). Similar results were obtained in ONS-76 cells (Supplementary Figure S1). Scratch-wound assay (for migration) and transwell invasion assay (for invasion) were used to investigate the effect of metformin on the Daoy cell migration and invasion. Compared with the control group, the cellular migration and invasive capacity decreased with the increased metformin concentration and prolonged treatment time (Figures 2A–D).

### Metformin promoted apoptosis of the Shh subgroup MB cell line

**FIGURE 3:**
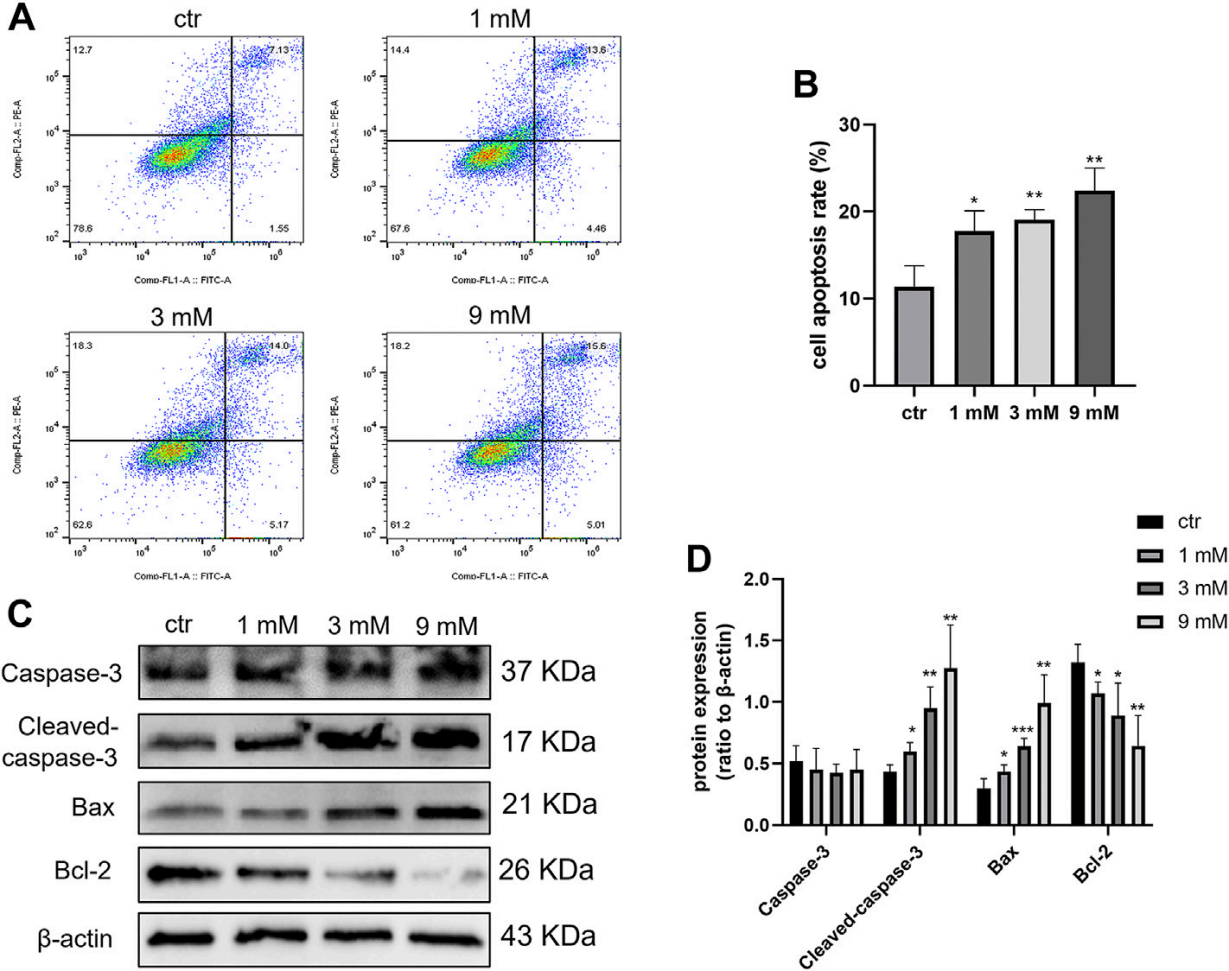
Cell apoptosis was induced by metformin treatment on Daoy cells. **(A,B)** Flow cytometry was used to analyze apoptosis on Daoy cells after 24 h of metformin treatment (*n* = 3), Annexin V-FITC-positive cells (Q2 + Q4) were considered as apoptotic. **(C,D)** Apoptosis-associated proteins were analyzed using western blot analysis. The relative Bcl-2, Bax, Caspase-3, and Cleaved-caspase-3 were normalized to that of β-actin (*n* = 4). Data are presented as the mean ± SD, t-tests were used to determine the significance. *p < 0.05; **p < 0.01; ***p < 0.001 compared with the control group.

To determine the apoptosis effect of metformin on Daoy cells, cell apoptosis was detected by flow cytometry. After treating Daoy cells with metformin (0, 1, 3, and 9 mm) for 24 h, the percentage of apoptotic Daoy cells elevated with the increase of metformin concentration (Figures 3A and B). In western blot, metformin inhibited the expression level of Bcl-2 and promoted the levels of Bax and Cleaved-caspase-3 in Daoy cells but had no significant effect on Caspase-3 (Figures 3C and D). These results indicated that metformin induced the apoptosis of Daoy cells.

### Metformin arrested cell cycle of the Shh subgroup MB cell line

**FIGURE 4:**
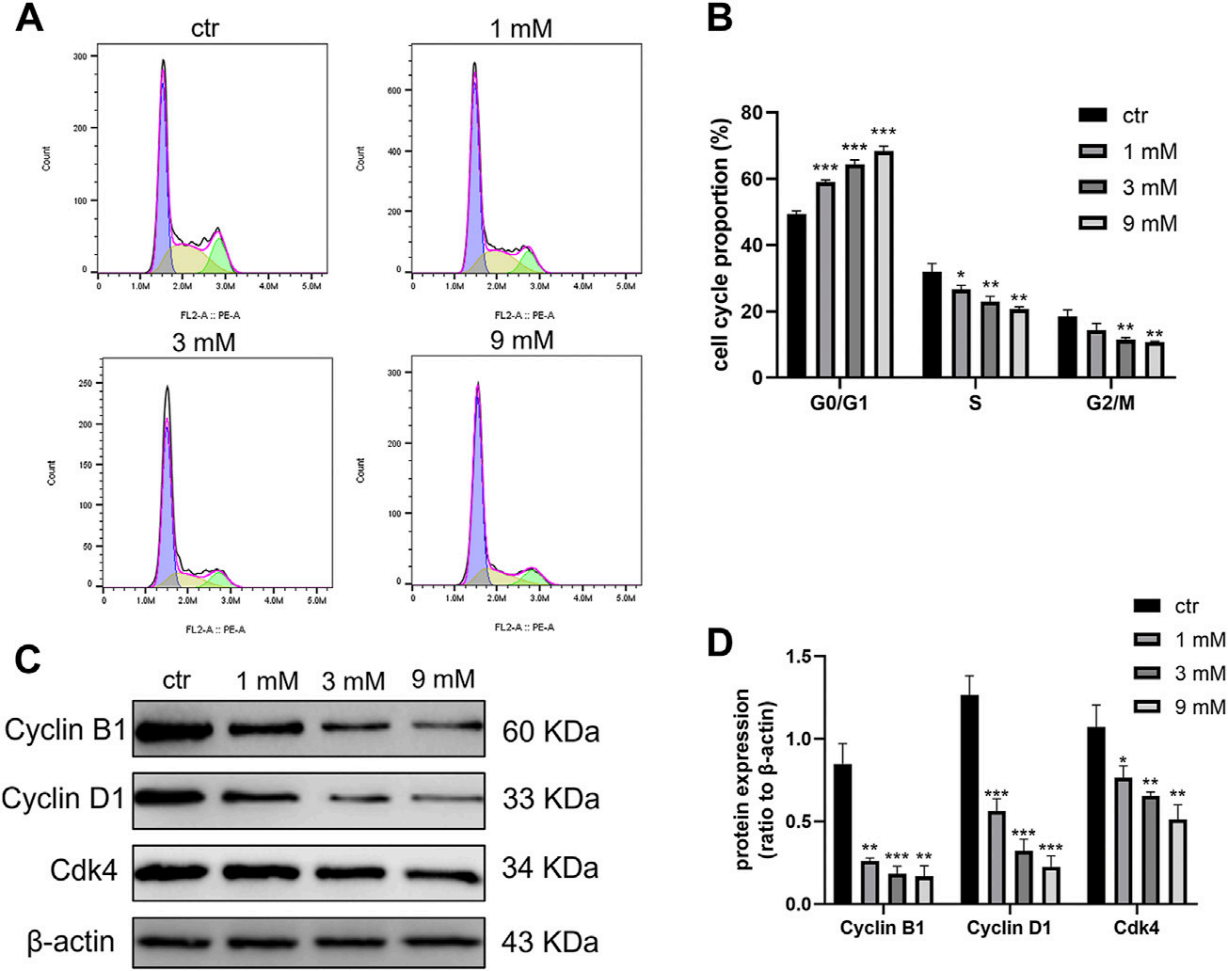
Cell cycle was blocked at the G0/G1 phase by metformin treatment on Daoy cells. **(A,B)** Flow cytometry was used to detect cell cycle distribution on Daoy cells after 24 h of metformin treatment (*n* = 3). **(C,D)** The proteins associated with the cell cycle were analyzed using western blot analysis. The relative Cyclin B1, Cyclin D1, and Cdk4 levels were normalized to that of β-actin (*n* = 4). Data are presented as the mean ± SD, t-tests were used to determine the significance. *p < 0.05; **p < 0.01; ***p < 0.001 compared with the control group.

To investigate the effect of metformin on the Daoy cell cycle, the cell cycle was detected by flow cytometry. After treating Daoy cells with metformin (0, 1, 3, and 9 mm) for 24 h, the percentage of cells in the G0/G1 phase was increased, and the rate of cells in the S and G2/M phase was decreased (Figures 4A and B). In addition, the expression of key G1-phase proteins Cyclin D1, CDK4, and the mitosis-related protein Cyclin B1 decreased (Figures 4C and D). These results indicated that metformin arrested the cell cycle of Daoy cells in the G0/G1 phase.

### Metformin suppressed the Shh signaling pathway through AMPK in the Shh subgroup MB cell line

**FIGURE 5:**
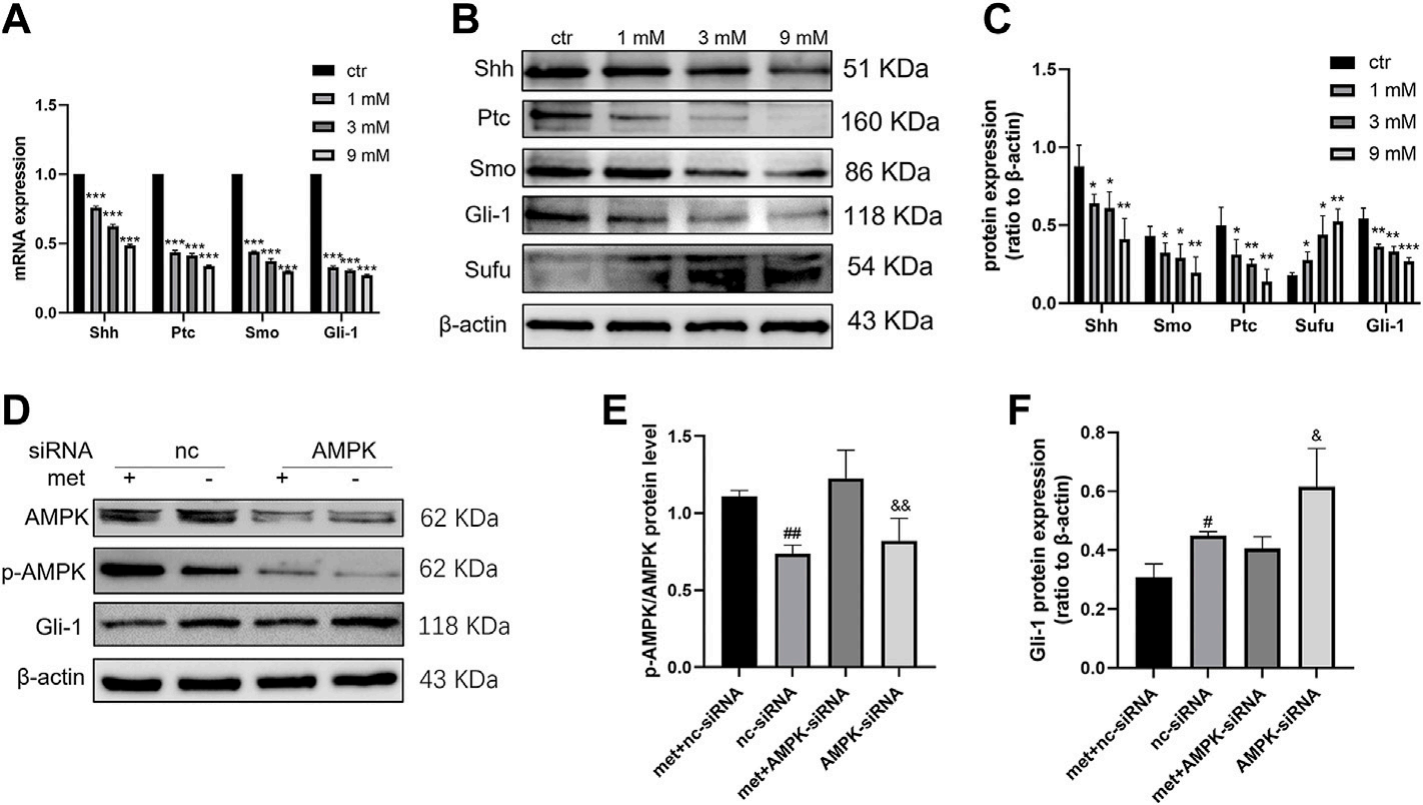
Metformin inhibited the expression of the Shh signaling pathway in Daoy cells. **(A)** The mRNA levels of Shh, Smo, Ptc, and Gli-1 were analyzed using quantitative PCR after 24 h of metformin treatment; GAPDH served as a control (n = 3, t-tests were used to determine the significance). **(B,C)** The protein levels of Shh, Smo, Ptc, Sufu, and Gli-1 were analyzed using western blot analysis after metformin treatment, β-actin served as a control (*n* = 4, t-tests were used to determine the significance). **(D–F)** The protein levels of AMPK, p-AMPK (Thr172), and Gli-1 were analyzed by western blot after AMPK siRNA and metformin (3 mM) treatment for 24 h, β-actin served as a control (*n* = 3, one-way ANOVA multiple comparisons were used to determine the significance). Data are presented as the mean ± SD. *p < 0.05; **p < 0.01; ***p < 0.001 compared with the control group. #*p* < 0.05; ##*p* < 0.01 compared with the met + nc-siRNA group. *P* < 0.05; and *p* < 0.01 compared with the met + AMPK-siRNA group.

To determine the effects of metformin on Shh signaling and whether this effect is exerted by activating AMPK. After treating Daoy cells with metformin (0, 1, 3, and 9 mm) for 24 h, the mRNA and protein levels of Shh, Smo, Ptc, and Gli-1 decreased in a dose-dependent manner following treatment with metformin (Figures 5A–C). To verify the role of AMPK in regulating the expressions of Shh signaling pathway proteins, we treated Daoy cells with AMPK siRNA for 24 h and then treated them with metformin (3 mm) for another 24 h. The results showed that metformin upregulated the phosphorylation level of AMPK (Thr172) (p-AMPK). In addition, the effect of metformin on reducing Gli-1 expression could be reversed by AMPK siRNA (Figure 5D). These results indicated that the molecular mechanism by which metformin suppressed Shh signaling pathway might involve the activation of AMPK.

### The antitumor effect of metformin on MB partly depended on the Shh pathway

**FIGURE 6:**
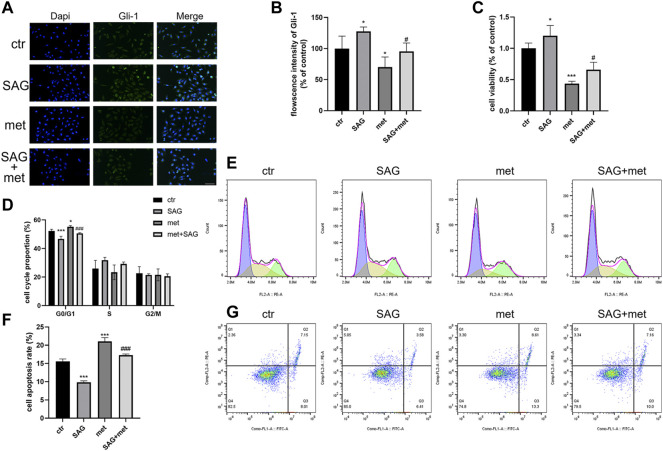
Metformin exerted antitumor effect on Daoy cells through the Shh signaling pathway. **(A,B)** The cells were treated with SAG (100 nm) for and metformin (3 mm) for 24 h and assessed by immunofluorescence assay with an anti-Gli-1 antibody (scale bar, 50 μm). **(C)** The cells were treated with SAG (100 nm) and metformin (3 mm) for 24 h, and the viability of cells were tested by CCK-8 assay. **(D–G)** Flow cytometry was used to analyze apoptosis and cell cycle distribution on Daoy cells after SAG (100 nm) for and metformin (3 mm) for 24 h. Data are presented as the mean ± SD, one-way ANOVA multiple comparisons were used to determine the significance. *p < 0.05; **p < 0.01; ***p < 0.001 compared with the control group. #*p* < 0.05; ##*p* < 0.01; ###*p* < 0.001 compared with the met group.

**FIGURE 7:**
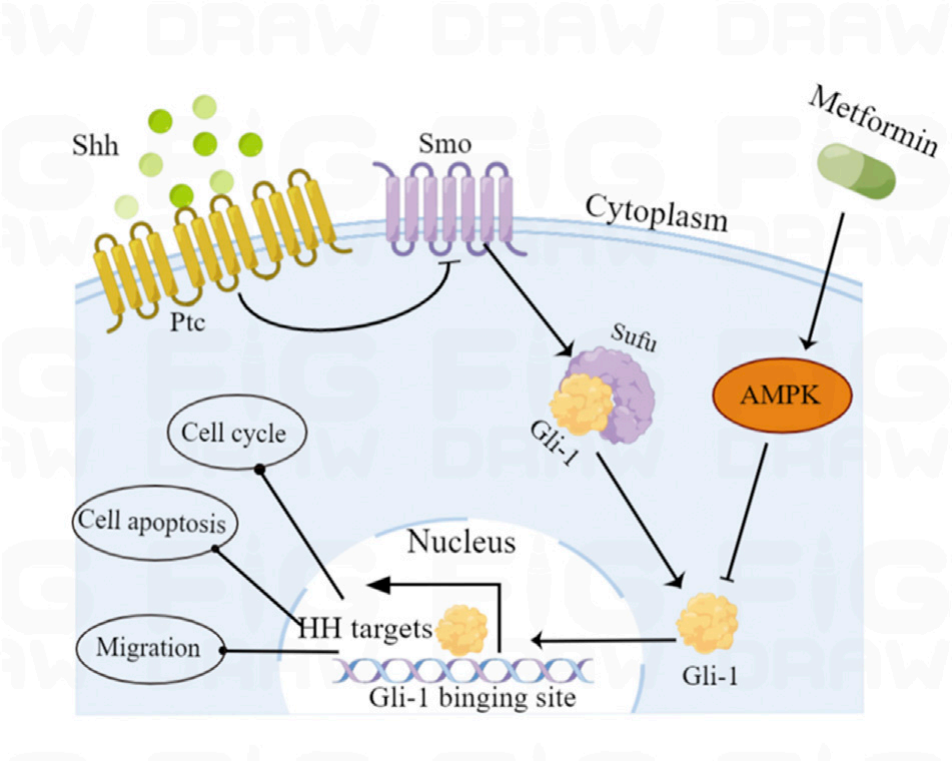
Schematic diagram of the proposed molecular mechanism by which metformin exerts anticancer effects *via* AMPK-mediated inhibition of the Shh signaling pathway in medulloblastoma.

To explore whether the Shh pathway mediates the antitumor effect of metformin on medulloblastoma, after the treatment of Daoy cells with SAG (100 nm) and metformin (3 mm), we found that the SAG can attenuate the inhibitory effect of metformin on Gli-1 (Figures 6A and B). In the detection of cell proliferation viability, SAG also reduced the inhibitory effect of metformin on cell viability (Figure 6C). This effect may be related to apoptosis and cell cycle arrest. We found that SAG can reduce the pro-apoptotic and cell cycle arrest effects of metformin on Daoy cells (Figures 6D–G). These results suggested that the Shh signaling pathway mediated the anti-proliferation effect of metformin on the Daoy cells by participating in apoptosis and cell cycle (Figure 7).

### Metformin resisted Shh subgroup MB growth *in vivo*

**FIGURE 8:**
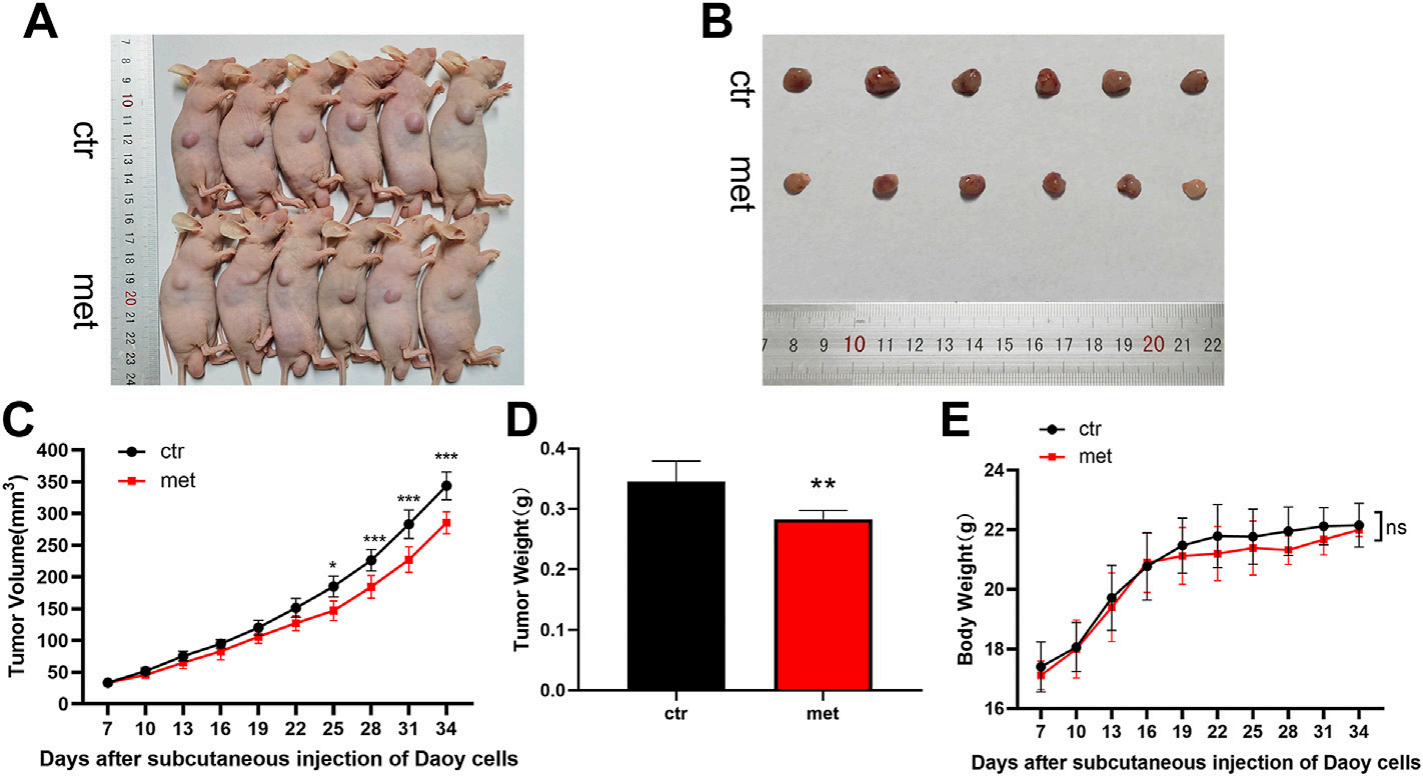
Metformin resisted Shh subgroup MB growth *in vivo*. **(A)** Photo of the nude mice bearing subcutaneous xenograft tumor. **(B)** Photo of tumors removed from mice after 24 days of metformin (200 mg/kg) or PBS treatment. **(C)** Tumor volume was recorded every 3 days **(D)**The weight of the excised tumor tissue. **(E)** Body weight of mice was recorded every 3 days (*n* = 6). Data are presented as the mean ± SD, two-way ANOVA multiple comparisons were used to determine the significance. *p < 0.05; **p < 0.01; ***p < 0.001 compared with the control group.

We conducted animal experiments to explore the anticancer effect of metformin on Shh subgroup MB *in vivo*. Oral administration of 300 μL metformin (200 mg/kg) or an equivalent volume of PBS was adopted after the subcutaneous formation of the tumor using Daoy cells. After treatment for 24 days, the mice in the metformin group demonstrated reduced tumor volumes and reduction in excised tumor weights when compared to the mice in the control group (Figures 8A–D). During the experiment, there was no significant difference in body weight between the two groups of mice (Figure 8E).

## Discussion

Most Shh subgroup MBs contain germline or somatic mutations in Shh signaling pathway-related genes, resulting in the activation of Shh signaling and promoting tumor progression (Cotter and Hawkins, 2022). Commonly mutated or deleted genes included Ptc (43%) and Sufu (10%); in addition, Smo (9%) mutations, Gli-1/2 (9%), and N-myc (7%) amplifications were sometimes observed (Northcott et al., 2017). Early studies on Smo antagonists showed a good inhibitory effect on the Shh pathway. Unfortunately, drug resistance occurs due to mutations in SMO and its downstream genes (Yauch et al., 2009; Ocasio et al., 2019). Therefore, multi-target combination therapy may be a necessary means to effectively control the disease. Metformin is a biguanide semi-synthetic oral hypoglycemic drug, mainly used for the treatment of type 2 diabetes. In 2005, Evans JM et al. first found that metformin reduces the incidence of tumors in patients with type 2 diabetes, and a series of related basic and clinical studies have been carried out since then (Evans et al., 2005; Higurashi et al., 2016; Munoz et al., 2021). In the present study, we found that metformin exhibited anticancer activity in MB by inhibiting cell proliferation, migration, and invasion in a dose-and-time-dependent manner *in vitro*. Moreover, metformin also showed an inhibitory effect on MB growth *in vivo* experiments. Among them, metformin inhibited cell proliferation by regulating cell cycle and apoptosis. Crucially, we found that metformin inhibited Shh signaling pathway in MB, and AMPK mediated part of this effect.

As a programmed cell death, apoptosis plays a key role in cancer therapy. Caspase-3 is an important protease in the process of apoptosis, and its activated form is involved in DNA repair and cell proliferation (Sergeeva et al., 2017). Previous studies have shown that metformin has a dual effect on apoptosis in different diseases, both promoting and inhibiting apoptosis. In studies related to cardiomyopathy, metformin inhibits apoptosis by reducing the expression of Cleaved-caspase-3, thereby attenuating hyperhomocysteinemia-induced cardiac hypertrophy and cardiac fibrosis (Zhao et al., 2021). However, metformin can reduce mitochondrial oxidative phosphorylation and intracellular ATP content, activate AMPK, and increase apoptosis in breast cancer cells (Haugrud et al., 2014). In related studies of the Shh signaling pathway, Ptc can activate caspase-mediated apoptosis when thhe Shh signaling pathway is inhibited (Brennan et al., 2012; Sigafoos et al., 2021). This study shows that metformin induces apoptosis in Daoy cells. The results showed that Daoy cells treated with metformin showed a significant downregulation of the anti-apoptotic protein Bcl-2, while the pro-apoptotic protein Bax and Cleaved-caspase-3 increased. The results also showed that the mRNA and protein expression levels of the Shh pathway were significantly decreased in metformin-treated Daoy cells in a dose-dependent manner. To further investigate the correlation between the apoptotic effect of metformin and its inhibitory effect on the Shh signaling pathway, we treated cells with SAG (a specific activator of the Shh signaling pathway). We observed that SAG could reverse part of the apoptosis-promoting effects of metformin. These findings support a significant role in the inhibition of the Shh signaling pathway in metformin-induced apoptosis in Daoy cells.

Disorders of cell cycle regulation exist in the occurrence and development of most malignant tumors. Previous studies have shown that G0/G1 cell cycle arrest is a mechanism of metformin’s antitumor effect, which has been demonstrated in lung and kidney cancer cells (Jin et al., 2017; Xie et al., 2017). Regulation of the cell cycle is dependent on the action of a series of Cyclin-Cdk-CD inhibitors (Knudsen et al., 2022). These special complexes regulate each phase of the cell cycle. Cyclin D1/Cdk1-4 are vital proteins regulating the G1/S transition, while Cyclin B1 initiates mitosis by promoting the cell G2/M transition (Khan et al., 2022). The dysregulated expression of the cell cycle-related proteins plays an important role in the growth, differentiation, apoptosis, and metastasis of various tumor cells (Montalto and De Amicis, 2020). Western blot results of this study showed that Daoy cells treated with metformin showed a significant downregulation of the decreased Cyclin D1, Cdk4, and Cyclin B1 expression. This is consistent with our observation in flow cytometry that the cell cycle was arrested in the G0/G1 phase. The Shh pathway also plays an important role in regulating the cell cycle. Gli-1 can promote the transcription of Cyclin D1, and Ptc can phosphorylate Cyclin B1 to promote cell proliferation (Barnes et al., 2001; Sigafoos et al., 2021). In our study, SAG was able to partially reverse the cycle arrest effect of metformin on Daoy cells. These results suggested that the Shh signaling pathway is involved in metformin-mediated cell cycle arrest in Daoy cells.

Tumor invasion and metastasis are closely related to epithelial to mesenchymal transition (EMT). The occurrence of EMT results in the weakening of tight junctions between epithelial cells and the loss of epithelial cell polarity, thereby enhancing the motility of epithelial cells (Jinesh and Brohl, 2022). The Shh signaling pathway also played a role in promoting the occurrence of EMT. In pancreatic cancer studies, it was found that the Shh signaling pathway promotes the process of EMT by affecting the components of various signaling pathways, including TGFβ, Ras, Wnt, PI3K/AKT, Integrin, and S100A4 (Xu et al., 2012). The study of gastric cancer also found that the increase in tumor lung metastasis was related to the activation of EMT by the Shh signaling pathway (Yoo et al., 2011). This is consistent with our observation that metformin inhibited the migration and invasion of Daoy cells, and inhibition of the Shh signaling pathway may mediate this effect by reducing EMT activity.

AMPK is the most important downstream effector of metformin and has been found to mediate the anticancer effects of metformin in multiple tumor cell lines (Chen et al., 2021; Lu et al., 2021). However, the relationship between AMPK and the Shh pathway is not clear, some studies pointed out that activated AMPK inhibits SHH signaling by phosphorylating Gli-1 and degrading it (Li et al., 2015). In addition, AMPK has indirect regulatory effects on the Shh pathway mediated by mTOR, FoxO1, and GSK3β (Asha et al., 2020) (Sun et al., 2016). In the present study, we found that metformin reduced the expression levels of the Shh signaling pathway and increased the ratio of p-AMPK/AMPK in Daoy cells. Gli-1 is an important downstream effector of the Shh pathway, and its expression level was partially reversed by AMPK siRNA under the inhibitory effect of metformin. Moreover, with the reduction of Gli-1 expression, the expression of upstream molecules in the pathway was also suppressed. Interestingly, the expression of the pathway negative regulator Sufu was upregulated, which may be related to the NEK2A-mediated indirect inhibition of Sufu degradation by Gli-1. These results suggest that AMPK is involved in regulating the non-canonical Shh pathway in Daoy cells by metformin.

Our study showed that metformin inhibited the proliferation, migration, and invasion of Shh subtype MB and promoted apoptosis and cell cycle arrest. The AMPK/Shh signaling pathway mediates part of this tumor suppressor effect. However, the concentration of metformin in cell culture (1–9 mm) is much higher than the plasma drug concentration (5–25 µm) of patients (Dowling et al., 2016). In order to explore the rationality of metformin as an anticancer drug, we established a medulloblastoma xenograft mouse model and administered it metformin orally at 200 mg/kg/day. According to the Reagan–Shaw formula (Reagan-Shaw et al., 2008), the human equivalent of the murine dose of 200 mg/kg is 973 mg in an average-sized human (60 kg), while the standard human therapeutic concentration of metformin is 1,000–2,500 mg/day. Satisfyingly, this dose of metformin effectively inhibited medulloblastoma growth and had no significant effect on growth in mice. These results suggest that metformin may be a potential chemotherapeutic agent for Shh-type medulloblastoma. Nevertheless, the anticancer activity of such doses in mice and the possibility of achieving comparable levels in humans by rationally increasing the dose suggest a reassessment of metformin dosing regimens in anticancer treatment to optimize plasma drug levels and delivery to the tumor. In future studies, we need to fully characterize the mechanism of action of metformin at effective antitumor concentrations and evaluate its efficacy as a viable anticancer therapy.

## Data Availability

The original contributions presented in the study are included in the article/Supplementary Material; further inquiries can be directed to the corresponding authors.

## References

[B1] Amayiri N. Swaidan M. Ibrahimi A. Hirmas N. Musharbash A. Bouffet E. (2021). Molecular subgroup is the strongest predictor of medulloblastoma outcome in a resource-limited country. JCO Glob. Oncol. 7, 1442–1453. 10.1200/GO.21.00127 34609903 PMC8492378

[B2] Asha K. Balfe N. Sharma-Walia N. (2020). Concurrent control of the kaposi's sarcoma-associated herpesvirus life cycle through chromatin modulation and host hedgehog signaling: A new prospect for the therapeutic potential of lipoxin A4. J. Virol. 94 (9), e021777-19. 10.1128/JVI.02177-19 PMC7163125 32102879

[B3] Azatyan A. Zhang S. Darabi A. Siesjö P. Wang T. Zaphiropoulos P. (2021). Circular RNAs in hedgehog signaling activation and hedgehog-mediated medulloblastoma tumors. Cancers 13 (20), 5138. 10.3390/cancers13205138 34680287 PMC8533754

[B4] Barnes E. Kong M. Ollendorff V. Donoghue D. (2001). Patched1 interacts with cyclin B1 to regulate cell cycle progression. EMBO J. 20 (9), 2214–2223. 10.1093/emboj/20.9.2214 11331587 PMC125436

[B5] Brennan D. Chen X. Cheng L. Mahoney M. Riobo N. (2012). Noncanonical hedgehog signaling. Vitam. Horm. 88, 55–72. 10.1016/B978-0-12-394622-5.00003-1 22391299 PMC3513281

[B6] Chatterjee A. Maitre M. Dasgupta A. Sridhar E. Gupta T. (2022). Multidisciplinary management of medulloblastoma: Consensus, challenges, and controversies. Methods Mol. Biol. 2423, 215–235. 10.1007/978-1-0716-1952-0_19 34978701

[B7] Chen C. Wang H. Geng X. Zhang D. Zhu Z. Zhang G. (2021). Metformin exerts anti-AR-negative prostate cancer activity via AMPK/autophagy signaling pathway. Cancer Cell Int. 21 (1), 404. 10.1186/s12935-021-02043-2 34399755 PMC8369631

[B8] Cotter J. Hawkins C. (2022). Medulloblastoma: WHO 2021 and beyond. Pediatr. Dev. Pathol. 25 (1), 23–33. 10.1177/10935266211018931 35168417

[B9] de Marañón A. Díaz-Pozo P. Canet F. Díaz-Morales N. Abad-Jiménez Z. López-Domènech S. (2022). Metformin modulates mitochondrial function and mitophagy in peripheral blood mononuclear cells from type 2 diabetic patients. Redox Biol. 53, 102342. 10.1016/j.redox.2022.102342 35605453 PMC9124713

[B10] Dowling R. Lam S. Bassi C. Mouaaz S. Aman A. Kiyota T. (2016). Metformin pharmacokinetics in mouse tumors: Implications for human therapy. Cell Metab. 23 (4), 567–568. 10.1016/j.cmet.2016.03.006 27076069

[B11] Evans J. Donnelly L. Emslie-Smith A. Alessi D. Morris A. (2005). Metformin and reduced risk of cancer in diabetic patients. BMJ Clin. Res. ed) 330 (7503), 1304–1305. 10.1136/bmj.38415.708634.F7 PMC558205 15849206

[B12] Fan C. Wang Y. Liu Z. Sun Y. Wang X. Wei G. (2015). Metformin exerts anticancer effects through the inhibition of the Sonic hedgehog signaling pathway in breast cancer. Int. J. Mol. Med. 36 (1), 204–214. 10.3892/ijmm.2015.2217 25999130 PMC4494591

[B13] Haugrud A. Zhuang Y. Coppock J. Miskimins W. (2014). Dichloroacetate enhances apoptotic cell death via oxidative damage and attenuates lactate production in metformin-treated breast cancer cells. Breast Cancer Res. Treat. 147 (3), 539–550. 10.1007/s10549-014-3128-y 25212175 PMC4184194

[B14] Heckman-Stoddard B. DeCensi A. Sahasrabuddhe V. Ford L. (2017). Repurposing metformin for the prevention of cancer and cancer recurrence. Diabetologia 60 (9), 1639–1647. 10.1007/s00125-017-4372-6 28776080 PMC5709147

[B15] Higurashi T. Hosono K. Takahashi H. Komiya Y. Umezawa S. Sakai E. (2016). Metformin for chemoprevention of metachronous colorectal adenoma or polyps in post-polypectomy patients without diabetes: A multicentre double-blind, placebo-controlled, randomised phase 3 trial. Lancet. Oncol. 17 (4), 475–483. 10.1016/S1470-2045(15)00565-3 26947328

[B16] Jin D. Kim Y. Lee B. Han J. Kim H. Shim Y. (2017). Metformin induces cell cycle arrest at the G1 phase through E2F8 suppression in lung cancer cells. Oncotarget 8 (60), 101509–101519. 10.18632/oncotarget.21552 29254182 PMC5731892

[B17] Jinesh G. Brohl A. (2022). Classical epithelial-mesenchymal transition (EMT) and alternative cell death process-driven blebbishield metastatic-witch (BMW) pathways to cancer metastasis. Signal Transduct. Target. Ther. 7 (1), 296. 10.1038/s41392-022-01132-6 35999218 PMC9399134

[B18] Khan H. Alam W. Alsharif K. Aschner M. Pervez S. Saso L. (2022). Alkaloids and colon cancer: Molecular mechanisms and therapeutic implications for cell cycle arrest. Mol. (Basel, Switz. 27 (3), 920. 10.3390/molecules27030920 PMC8838632 35164185

[B19] Kim Y. Kim S. Cho S. Park J. Choi I. Lee Y. (2014). Long-term metformin use reduces gastric cancer risk in type 2 diabetics without insulin treatment: A nationwide cohort study. Aliment. Pharmacol. Ther. 39 (8), 854–863. 10.1111/apt.12660 24612291

[B20] Knudsen E. Kumarasamy V. Nambiar R. Pearson J. Vail P. Rosenheck H. (2022). CDK/cyclin dependencies define extreme cancer cell-cycle heterogeneity and collateral vulnerabilities. Cell Rep. 38 (9), 110448. 10.1016/j.celrep.2022.110448 35235778 PMC9022184

[B21] Li Y. Luo J. Mosley Y. Hedrick V. Paul L. Chang J. (2015). AMP-activated protein kinase directly phosphorylates and destabilizes hedgehog pathway transcription factor GLI1 in medulloblastoma. Cell Rep. 12 (4), 599–609. 10.1016/j.celrep.2015.06.054 26190112 PMC4521589

[B22] Lu T. Li M. Zhao M. Huang Y. Bi G. Liang J. (2021). Metformin inhibits human non-small cell lung cancer by regulating AMPK-CEBPB-PDL1 signaling pathway. Cancer Immunol. Immunother. 71 (4), 1733–1746. 10.1007/s00262-021-03116-x 34837101 PMC10991609

[B23] Montalto F. De Amicis F. (2020). Cyclin D1 in cancer: A molecular connection for cell cycle control, adhesion and invasion in tumor and stroma. Cells 9 (12), E2648. 10.3390/cells9122648 33317149 PMC7763888

[B24] Munoz L. Huang L. Bommireddy R. Sharma R. Monterroza L. Guin R. (2021). Metformin reduces PD-L1 on tumor cells and enhances the anti-tumor immune response generated by vaccine immunotherapy. J. Immunother. Cancer 9 (11), e002614. 10.1136/jitc-2021-002614 34815353 PMC8611422

[B25] Northcott P. Buchhalter I. Morrissy A. Hovestadt V. Weischenfeldt J. Ehrenberger T. (2017). The whole-genome landscape of medulloblastoma subtypes. Nature 547 (7663), 311–317. 10.1038/nature22973 28726821 PMC5905700

[B26] Northcott P. Robinson G. Kratz C. Mabbott D. Pomeroy S. Clifford S. (2019). Medulloblastoma. Nat. Rev. Dis. Prim. 5 (1), 11. 10.1038/s41572-019-0063-6 30765705

[B27] Ocasio J. Babcock B. Malawsky D. Weir S. Loo L. Simon J. (2019). scRNA-seq in medulloblastoma shows cellular heterogeneity and lineage expansion support resistance to SHH inhibitor therapy. Nat. Commun. 10 (1), 5829. 10.1038/s41467-019-13657-6 31863004 PMC6925218

[B28] Park S. Willingham M. Qi J. Cheng S. (2018). Metformin and JQ1 synergistically inhibit obesity-activated thyroid cancer. Endocr. Relat. Cancer 25 (10), 865–877. 10.1530/ERC-18-0071 29914872 PMC6059993

[B29] Patel S. Bhatnagar A. Wear C. Osiro S. Gabriel A. Kimball D. (2014). Are pediatric brain tumors on the rise in the USA? Significant incidence and survival findings from the SEER database analysis. Childs Nerv. Syst. 30 (1), 147–154. 10.1007/s00381-013-2307-1 24162619

[B30] Reagan-Shaw S. Nihal M. Ahmad N. (2008). Dose translation from animal to human studies revisited. FASEB J. official Publ. Fed. Am. Soc. Exp. Biol. 22 (3), 659–661. 10.1096/fj.07-9574LSF 17942826

[B31] Sergeeva T. Shirmanova M. Zlobovskaya O. Gavrina A. Dudenkova V. Lukina M. (2017). Relationship between intracellular pH, metabolic co-factors and caspase-3 activation in cancer cells during apoptosis. Biochim. Biophys. Acta. Mol. Cell Res. 1864 (3), 604–611. 10.1016/j.bbamcr.2016.12.022 28063999

[B32] Sigafoos A. Paradise B. Fernandez-Zapico M. (2021). Hedgehog/GLI signaling pathway: Transduction, regulation, and implications for disease. Cancers 13 (14), 3410. 10.3390/cancers13143410 34298625 PMC8304605

[B33] Skowron P. Farooq H. Cavalli F. Morrissy A. Ly M. Hendrikse L. (2021). The transcriptional landscape of Shh medulloblastoma. Nat. Commun. 12 (1), 1749. 10.1038/s41467-021-21883-0 33741928 PMC7979819

[B34] Sun Y. Tao C. Huang X. He H. Shi H. Zhang Q. (2016). Metformin induces apoptosis of human hepatocellular carcinoma HepG2 cells by activating an AMPK/p53/miR-23a/FOXA1 pathway. Onco. Targets. Ther. 9, 2845–2853. 10.2147/OTT.S99770 27274280 PMC4869652

[B35] Xie W. Wang L. Sheng H. Qiu J. Zhang D. Zhang L. (2017). Metformin induces growth inhibition and cell cycle arrest by upregulating MicroRNA34a in renal cancer cells. Med. Sci. Monit. 23, 29–37. 10.12659/msm.898710 28045889 PMC5226302

[B36] Xu X. Zhou Y. Xie C. Wei S. Gan H. He S. (2012). Genome-wide screening reveals an EMT molecular network mediated by Sonic hedgehog-Gli1 signaling in pancreatic cancer cells. PloS one 7 (8), e43119. 10.1371/journal.pone.0043119 22900095 PMC3416762

[B37] Yauch R. Dijkgraaf G. Alicke B. Januario T. Ahn C. Holcomb T. (2009). Smoothened mutation confers resistance to a Hedgehog pathway inhibitor in medulloblastoma. Sci. (New York, NY) 326 (5952), 572–574. 10.1126/science.1179386 PMC5310713 19726788

[B38] Yeole U. Hegde S. Gothwal M. Prabhuraj A. Somanna S. Thennarasu K. (2021). What happens after therapy? Quality of life and neurocognitive functions of children with malignant posterior fossa tumors after adjuvant therapy. Neurol. India 69 (5), 1293–1301. 10.4103/0028-3886.329599 34747802 PMC7613140

[B39] Yoo Y. Kang M. Lee H. Kim B. Park J. Kim H. (2011). Sonic hedgehog pathway promotes metastasis and lymphangiogenesis via activation of Akt, EMT, and MMP-9 pathway in gastric cancer. Cancer Res. 71 (22), 7061–7070. 10.1158/0008-5472.CAN-11-1338 21975935

[B40] Zhao Q. Song W. Huang J. Wang D. Xu C. (2021). Metformin decreased myocardial fibrosis and apoptosis in hyperhomocysteinemia -induced cardiac hypertrophy. Curr. Res. Transl. Med. 69 (1), 103270. 10.1016/j.retram.2020.103270 33268288

