# Supplementary material for: Effects of metformin on Sonic hedgehog subgroup medulloblastoma progression: In vitro and in vivo studies
Source: Front Pharmacol. 2022 Oct 7;13:928853. doi: 10.3389/fphar.2022.928853 (PMC9585190; doi:10.3389/fphar.2022.928853)
Supplement: Supplementary file 1 [file DataSheet1.docx]

Supplementary Material

## Supplementary Figures


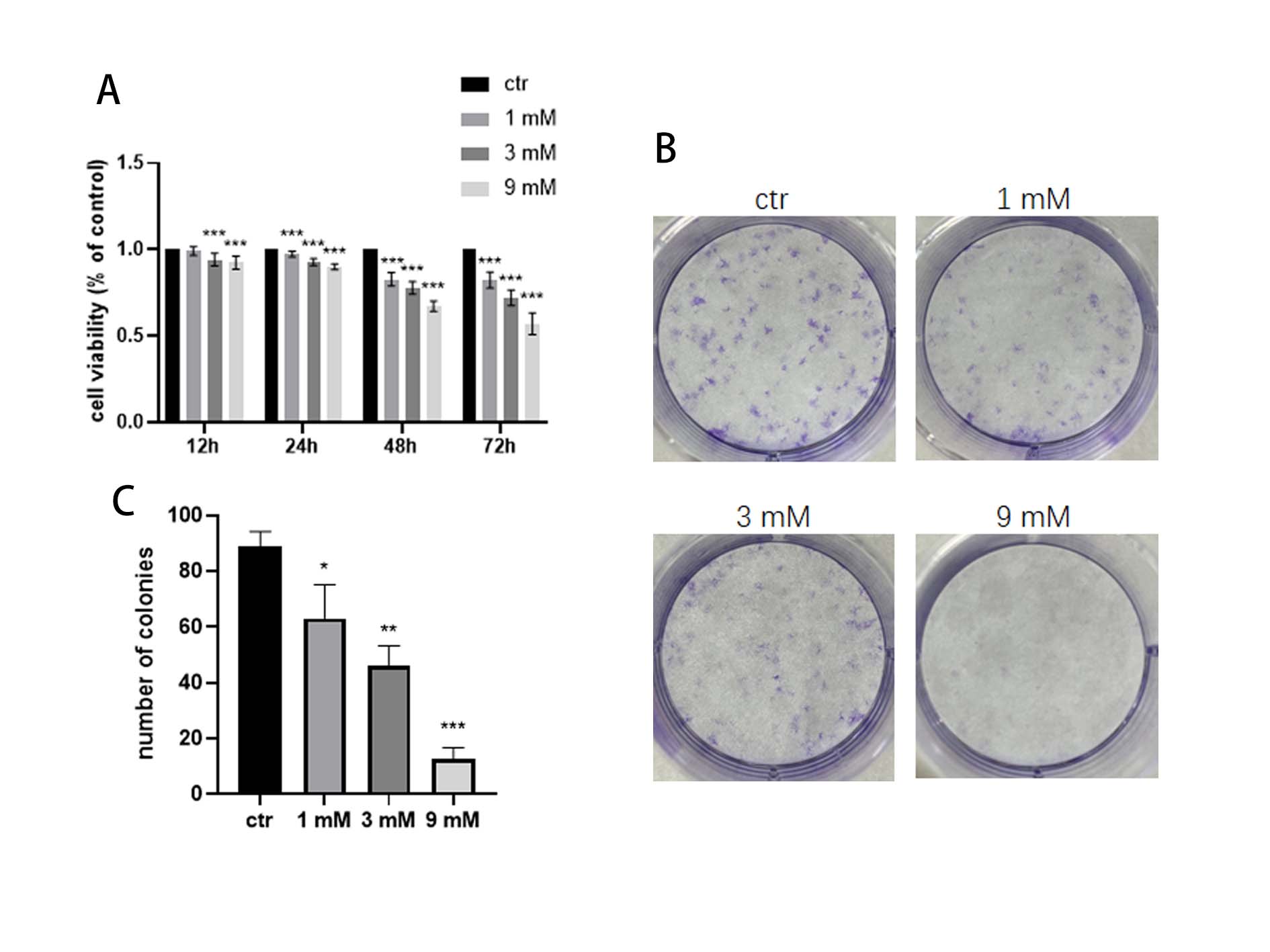


**Supplementary Figure 1.** Metformin inhibited the proliferation of ONS-76 cells*.* **(A)** Cck‑8 assay was used to investigate the effects of metformin treatment on cell viability. **(B, C)** Colony formation assay was used to detected the effects of metformin treatment on clone ability of cells. Data are presented as the mean ± SD. **p* < 0.05; ***p* < 0.01; ****p* < 0.001 compared with the control group.
